# Individualized Apartment Accommodation for People With Intellectual Disability: Protocol for a Qualitative Study Examining the Well-Being and Support Outcomes Linking Housing and Health

**DOI:** 10.2196/18248

**Published:** 2020-08-07

**Authors:** Phillippa Carnemolla

**Affiliations:** 1 School of Built Environment Faculty of Design Architecture and Building University of Technology Sydney Sydney Australia

**Keywords:** POE, postoccupancy evaluation, health and well-being, disability, housing, disability housing, intellectual disability, built environment, allied health care, disability support, group home, community support, community health, qualitative, autonomy

## Abstract

**Background:**

Understanding the outcomes associated with both receiving and providing support to people with intellectual disability in specific settings can facilitate the alignment of health providers, community care providers, architects, and urban planners to strengthen levels of autonomy and community participation of people with intellectual disability living in the community. This study explores the impact of providing support (available 24 hours a day) for people with intellectual disability in a high-density apartment. It seeks the perspectives of people with intellectual disability who have moved into an apartment from a group home (where 4-6 people with disability live), their families, and support staff. It will enable comparison between two models of supported accommodation, group homes and individualized apartments, in a community setting.

**Objective:**

The aims of this study are to explore the impact of an individualized apartment model of supported accommodation in a high-density setting on the well-being, autonomy, and participation of people with intellectual disability living and receiving support; the experience of providing care or support; and how this setting impacts the logistics of how quality support is provided.

**Methods:**

Qualitative research methods were employed as the primary means of collecting and analyzing data. There are two main sources of data in this study: (1) semistructured interviews with participants in up to 3 waves (pre, post 1, and post 2) and (2) pre- and postoccupancy evaluation data on the design, layout, and location details of the built environments. Coded interview data will be paired with pre- and postoccupancy evaluations of the two accommodation settings.

**Results:**

As of May 2020, we have recruited 55 participants. There have been 96 interviews conducted in 2 waves with people who have moved into supported accommodation, families, and staff. Collected data are currently being analyzed. We expect the results of the trial to be published in a peer-reviewed journal in late 2020.

**Conclusions:**

This paper sets out a study of an alternative housing and support model for people with intellectual disability. It will capture personal experiences of people with intellectual disability receiving support in an apartment compared to their experiences in a group home. It will also capture the experiences of support staff working in the new setting and reveal how this differs from a group home setting. The inclusion of pre (group home) and post (apartment integrated into a community setting) measures addresses evaluative and comparative questions around the nature and impacts of the small-scale apartment and support model for both those who live and receive support, and those who support them.

**International Registered Report Identifier (IRRID):**

DERR1-10.2196/18248

## Introduction

### Beyond the Group Home and Into an Apartment

The introduction of consumer-led health and disability funding across the world (including the United States, the United Kingdom, parts of Europe, and Australia) has changed in how disability housing support is provided and brought with it the opportunity for individualized living plans and accommodation settings. Developments in research across health and urban planning fields recognize the ways that urbanized, high-density settings influence a population’s health, well-being, and participation in growing cities [1,2]. People with intellectual disability have largely been excluded from these discussions, and community urban life in general. This exclusion can be explained to a large degree by the fact that, prior to consumer-led disability services, people with intellectual disability who received support in their daily life activities, were historically required to live in institutional care settings. In more recent years, since deinstitutionalization, people with intellectual disability have tended to live in congregate care housing such as group homes, which, because of their spatial footprint and number of bedrooms, are likely to be located in suburban settings.

People with intellectual disability have a right to equal choice, freedom, and control over their living arrangements, including where they live, who they live with, and who provides support to them. Despite the strong evidence linking housing type, design and location, support practices, and health outcomes [3-5], there is little evidence upon which community housing providers, health providers, and urban policy makers can make aligned decisions about planning, design, health, and support practices for people with intellectual disability receiving high support.

Supported accommodation performs not only as a place where people live but also as a place where personal and social support, and health care are coordinated, provided, and received. For people with intellectual disability who receive 24-hour support, group homes remain the predominant community-based, long-term accommodation option outside the family home [6,7]. A growing body of evidence has explored the outcomes of group homes as an accommodation model for people with intellectual disability and has recognized that the quality-of-life outcomes for those living and receiving support in group homes are highly variable [7,8]. Some of the expected improvements in the outcomes and lives of people with intellectual disability in the years of post-deinstitutionalization have not been realized, most notably the fact that community participation has not increased despite group homes being community based [8-10]. In addition to this, the power dynamics that the home and work duality of the group home environment brings (being both a home for a person with disability and a workplace for support staff) can result in less-than-homely environments and workplace cultures determining the household routines and activities [7,11,12]. The lack of homeliness of some group homes has also been highlighted by Robertson et al [13].

Increasing the diversity of accommodation models that benefit both the quality of life in those living and receiving support in the community, as well as ensuring the quality and sustainability of community health care provision, is an important area of public health research. There is evidence to suggest that alternatives to group homes are limited, with many young people with disability residing in aged care facilities [14], and a lack of choice within the housing market for supported accommodation is a problem across the world [15,16].

### Group Homes Versus Smaller Models of Supported Accommodation

Group homes are defined as “accommodation for between four and six people, where extensive or pervasive paid staff support is provided to the residents, both in the home and when leaving it to use community –based settings” [8]. The support is typically provided 24 hours of the day with support staff working a sleepover shift or awake shift throughout the night. Despite evidence indicating that smaller, community-based housing arrangements (1-2 people living and receiving support) result in better outcomes for people with intellectual disability, there has been comparatively little work undertaken to understand what living in, receiving support, and providing support in these smaller models in high-density settings mean. The research that has been undertaken in this area indicates that settings of 1-2 people living and receiving support result in better outcomes including more choice, self-determination, and freedom from staff [17-19].

The transition to self-directed disability funding such as the National Disability Insurance scheme in Australia and Self-Directed Support in the United Kingdom have enabled a wide variety of accommodation setting options and funding models where people with disability could live and receive high support [20]. Consistent with this trend, a not-for-profit housing and service provider in New South Wales, Australia (Provider) has implemented a model of smaller 1- or 2-bedroom apartments “salt and peppered” throughout a high-density, privately owned apartment development. The Provider case study will be the source of participants for this study. The Provider gives support to the apartments it owns and manages where people with intellectual disability live with 24-hour support. The apartments are distributed throughout a high-density apartment complex of 416 privately owned apartments.

Researchers in supported accommodation acknowledge that an array of factors including design, layout, location, size, and staffing impact the autonomy, independence, and well-being of adults with intellectual disability living in the community [10,17,21]. New housing models suitable for high-density city settings need to be evaluated to understand the health and support practice impacts of supported accommodation models at room, apartment, site, and neighborhood scales [2].

This research design will incorporate perspectives on the design of the built environment alongside perspectives of sense of home, quality of life, and participation outcomes for people with intellectual disability. Evaluating the impact of a change in dwelling location and design, such as changes to the size, density type, accessibility, community immersion, and other functional features of housing, as well as the nature of the individualized supported living plans will in turn bring about changes in outcomes for people living with disability and the way support is provided. Better design of accommodation, in both its quality and accessibility are acknowledged as central to the efforts of supporting the health, independence, and autonomy of people with intellectual disability in the community [18].

## Methods

### Aim and Research Questions

The overall aim of the evaluation is to understand the impact of providing high support for people with intellectual disability in a high-density apartment from the perspectives of those who live and receive support there, their families and guardians, and the support staff who work within this model. The objectives of this study are to explore the impact of an individualized apartment model of supported accommodation in a high-density setting (including its design and location) on:

- Well-being, autonomy, and participation of people with intellectual disability living and receiving support
- The experience of providing care and support, and the logistics of how support is provided

Qualitative research methods were employed as the primary means of collecting and analyzing data to understand the impact of individualized apartments as a supported accommodation model for people with intellectual disability. There is also some quantitative analysis undertaken, including a statistical analysis of participants and quantitative data rising from postoccupancy evaluation data on the built environments.

**Figure 1:**
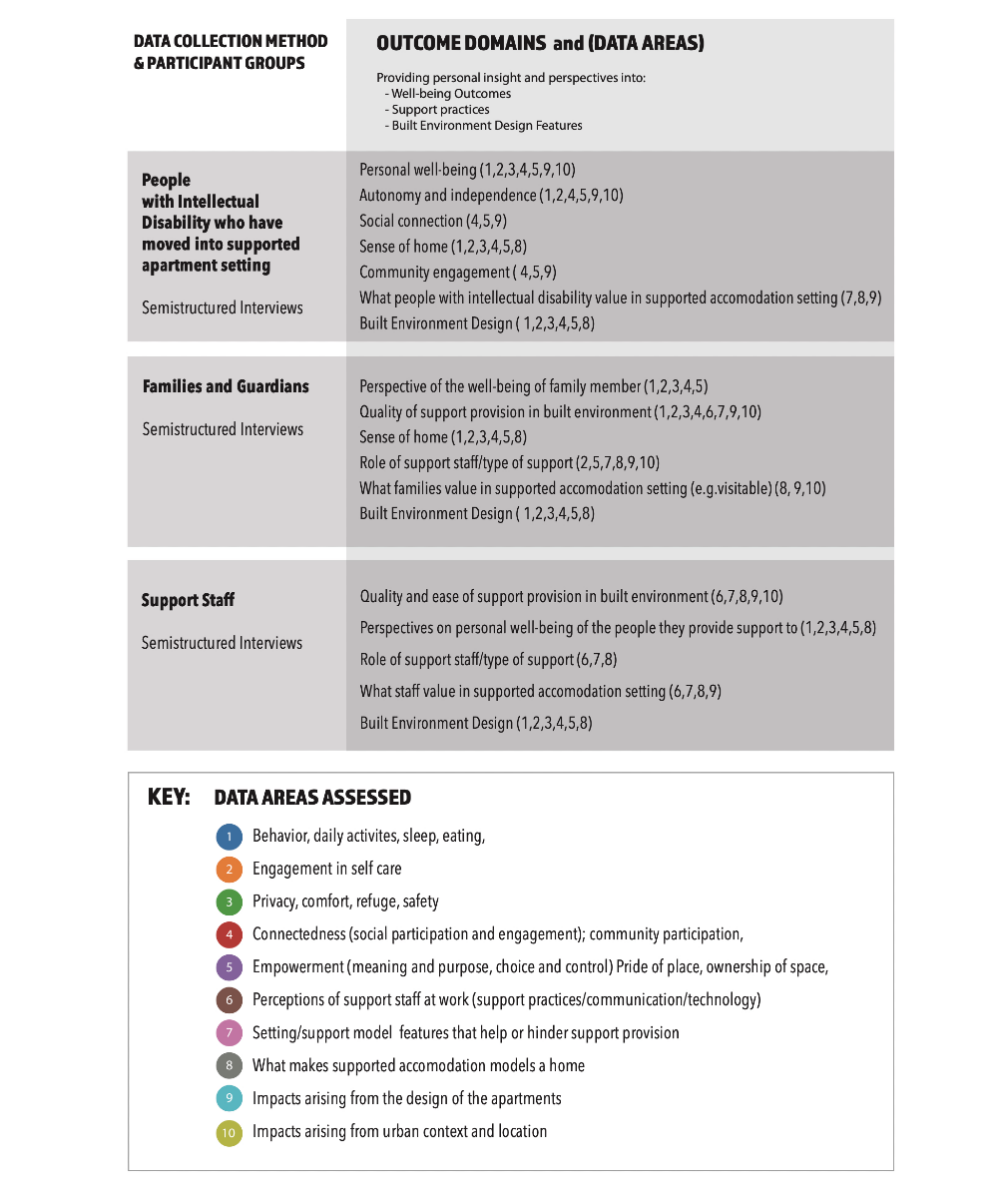
Approach to data collection.

There are two main sources of data in this study: (1) semistructured interviews will be conducted with participants in up to 3 waves (pre, post 1, and post 2) and (2) pre- and postoccupancy evaluation data [22] that captures design, layout, and location details of the built environments where support is provided or received. A summary of the data collection methods is provided in Figure 1.

For the qualitative part of the data collection, in-depth, semistructured interviews enable the researchers to explore the “deep meaning” and “inside view” that lie beneath the human behaviors and choices being explored in this research [23]. There are three main participant groups: people with intellectual disability who are living and receiving support in the accommodation, paid support staff, and families of people with intellectual disability. Up to 20 participants will be recruited from each participant group for this exploratory study, resulting in a total of up to 60 participants. The research applies a general inductive approach for analyzing the data, whereby meaning and concepts are primarily derived from the accounts of participants in the research [24-26].

As a mixed methodology, a qualitative approach coupled with postoccupancy data provides an opportunity to deepen understanding of the built environment’s influence on independence participation and support practices. The study is designed to build new evidence that informs researchers across disciplines (housing, disability, and community services) and begins to “make sense” of the relationships between housing design, location, and support systems that take place in a supported accommodation setting. Most importantly, the study provides an opportunity to represent the voice of people with intellectual disability by hearing their experiences of living in and receiving support in a new environment.

### Ethics Approval and Consent to Participate

Ethics approval was granted by the University of Technology Sydney (UTS) Human Research Ethics Committee approval number ETH17-2032: Supported Living Accommodation: Housing, Quality of Life and Support Services for people with intellectual disability. Participants (including people with intellectual disability, their families or guardians, and support staff) are required to sign a consent form to indicate their willingness to participate. Voluntary participation and the right to ask any questions, and to decline participation at any time, will be emphasized during the data collection. Easy read versions of Project Information Statements and consent forms were developed to inform participants with intellectual disabilities of the purpose and processes involved in the research.

### Setting and Participants

The Provider owns and gives supported accommodation for people with intellectual disability in 22 apartments (one or two bedroom) that are “salt-and-peppered” across a high-density, privately owned development of 416 apartments in Sydney, Australia. This apartment development has been selected as the setting for participant recruitment. Each apartment owned by the Provider has 1-2 bedrooms, providing 24-hour support to over 40 people with intellectual disability. The site has a number of apartment towers accessed by multiple lift wells and underground parking, and is secured by locked gates. The site has shared garden areas typical of a high-density development found in a larger city such as Sydney. Staff are rostered to provided 24 hour “awake” support across the 22 apartments located in all four towers on the development site. This is in contrast with the support provided previously in the group home settings, where 24-hour support was rostered with a “sleepover” shift overnight.

Self-selection sampling is to be used in this study to recruit participants living and receiving support in apartments owned by the Provider, their families, and support staff employed by the Provider. Posters explaining the research will be placed in staff quarters, and the researchers will attend family and staff meetings to explain the research aims. This study will target sampling at least 20 people with intellectual disability, 20 family carers, and 20 support staff.

The qualitative study will explore how the design of the home and accommodation setting influences the quality of life domains of the person receiving support and how it functions as a work environment for staff providing support. This will enable an understanding of different experiences and perspectives from three stakeholder groups:

1. People with intellectual disability who are living and receiving support in the new apartment, having moved from a group home
2. The families and guardians of people with intellectual disability
3. Support staff providing disability services in the supported apartment accommodation

The key elements of the qualitative design are outlined in Figure 1. Data collection methods include semistructured interviews conducted with people with intellectual disability, their families, and staff providing support. Semistructured interviews with people with disability and families will focus on understanding what aspects of the support model and environment design influence their quality of life and daily lives. The semistructured interviews with support staff will explore perspectives on how the built environment and supporting features and technologies impact their support provision, including identification of any current difficulties and perceptions of the new individualized model.

The data from this study will reveal how the different models of group home or individualized apartment living impact the lives of people with intellectual disability and the working practices of support staff. The quality of life approach will enable a comparison between the social impacts of living in a group home compared to individualized apartment accommodation models.

### Analysis

The research will gather data that combines housing design and experiences of people with intellectual disability, families, and support staff. Data analysis will explore how the design of the built environment intersects with outcomes for people with intellectual disability and support delivery, and the principles underpinning quality supported accommodation. The thematic analysis of the qualitative content will be undertaken using the stages reported by Green and colleagues [27], which includes immersion in the transcripts, text coding, creation of broader categories from the coded text, and identifying themes.

The coding process will involve both open and axial coding of the data and comparison of the dwellings (both group homes and apartments), with performance criteria developed from the literature. This methodological approach is based upon foundation work undertaken by Bridge and Donelly [28].

### Availability of Data and Material

Materials described in this paper pertain to the study protocol only and there are no raw data reported. The data sets are currently being collected and analyzed. The data sets generated or analyzed during this study are not publicly available due to the terms of consent that the participants agreed to but are available from the corresponding author on reasonable request.

## Results

As of May 2020, we have recruited 55 participants. There have been 96 interviews conducted in two waves with people who have moved in to supported accommodation, families, and staff. Collected data are currently being analyzed. We expect the results of the trial to be published in a peer-reviewed journal in late 2020 and early 2021.

## Discussion

### Receiving Support in Urbanized, High-Density Settings

This research will contribute to an evaluation of community care and disability housing models. The study will increase our understanding of the experiences of people with intellectual disability to inform person-led best practices for disability support in the community for people with disability. The study will also provide a means of understanding how support is influenced by the model of housing and will contribute to the body of knowledge about how urbanized, high-density settings influence health, well-being, and participation in growing cities.

By providing a comparison of group home models with individualized apartment accommodation, it is hoped that this research will also lead to better quality and more informed housing and community support choices for people with intellectual disability.

The theoretical framework that will be generated from this study will be practical and useful in producing knowledge about factors that influence independence, autonomy, support provision, and participation for people with intellectual disability. Pairing data with pre- and postoccupancy information about the design and location of the accommodation will enable specific environmental factors that influence support practice to be clearly identified. The framework will, therefore, be useful in guiding further support interventions and innovations that will address the needs of people with intellectual disability and the sustainability of the support workforce that underpins community-based supported accommodation models.

As housing availability and affordability is in decline in urbanized city centers around the world [29] and people with intellectual disability continue to experience layers of disadvantage [30], those with intellectual disability face limited opportunities to claim the right to make choices about where they live, who they live with, and who provides support to them and how. This research provides information upon which informed decisions can be made with and by people with intellectual disability so that they may live the life they choose to lead, participating and living in the communities of their choice.

### Limitations

The limitations of this particular study design include that the research is single arm (no control) and that participants are recruited from a single site; therefore, generalizability can be considered limited. This study can be considered exploratory with the potential to be broadened in scope and size to include other sites with similar accommodation models at a later date.

### Strengths

This research recognizes the role of environmental factors, including urban planning and housing design, in influencing the well-being, participation, and quality of life in people with intellectual disability. The accommodation design and setting also influences the type and quality of support provision provided to people with intellectual disability living in a community. The study will provide new insights into emerging health and well-being outcomes associated with community living for people with intellectual disability. It will also inform policies and practices for innovative, sustainable, and person-led models of high (24-hour) support provision in the community.

Despite being small in scale the study promises to lay the theoretical groundwork, produce policy learnings, and begin to build an evidence base that will be relevant for the disability sector in an area where changes to group homes as the default model have been difficult or slow to achieve. It will also establish an evidence base of associations between the design of the built environment and outcomes around support, participation, and independence for people with intellectual disability.

### Conclusion

This paper sets out a study of an alternative housing and support model for people with intellectual disability. The study will capture personal experiences of people with intellectual disability receiving high levels of support in an apartment compared to their experiences in a group home. It will also capture the experiences of support staff working in the new setting and reveal how this differs from providing support in a group home. The collected data will be triangulated with data from family and guardians’ perspectives.

The inclusion of pre (group home) and post (apartment integrated into a community setting) measures addresses evaluative and comparative questions around the nature and impacts of the small-scale apartment and support model for both those who live there and receive support, and those who support them.

## Appendix

Multimedia Appendix 1 Letter from government funder attesting to peer-reviewing of the funded project.

## Abbreviations

UTS: University of Technology Sydney
