# Supplementary material for: Individualized Apartment Accommodation for People With Intellectual Disability: Protocol for a Qualitative Study Examining the Well-Being and Support Outcomes Linking Housing and Health
Source: JMIR Res Protoc. 2020 Aug 7;9(8):e18248. doi: 10.2196/18248 (PMC7442944; doi:10.2196/18248)
Supplement: Multimedia Appendix 1 [file resprot_v9i8e18248_app1.pdf]

14 February 2020

National Disability Services

To whom it may concern

This letter is to confirm that the project research "Beyond the Group Home" was a successful recipient of the Innovative Workforce Fund in the First Grant Round which was administered in 2017.

The Innovative Workforce Fund was established by the Australian Commonwealth Government to encourage the development and sharing of innovative workforce practices to support rollout of the National Disability Insurance Scheme. The fund was administered by National Disability Services (NDS).

I confirm that all proposal applications underwent a rigorous peer-review process by an independent reference group set up by NDS specifically to assess the grant applications.

The research outlined in the successful application "Beyond the Group Home" was undertaken by the Chief Investigator, Dr Phillippa Carnemolla, at the University of Technology, Sydney in partnership with Achieve Australia Pty. Ltd.

The total amount of funds awarded was $130,000.

Kind regards

Gordon Duff
General Manager, Sector Development and Research
National Disability Services

Ph: 02 9256 3117
Fax: 02 9256 3123
Mob: 0428 694 374

Email: gordon.duff@nds.org.au
Web: www.nds.org.au

State Office:
Level 19, 66 Goulburn Street
Sydney NSW 2000

PO Box 20637
World Square NSW 2002

NDS acknowledges the Australian Aboriginal and Torres Strait Islander peoples as the first inhabitants of the nation and the Traditional Custodians of the lands where we live, learn and work.
